# Dilemma of Finding the Most Useful QTc Formula: A Retrospective Analysis of South-East London

**DOI:** 10.7759/cureus.99510

**Published:** 2025-12-17

**Authors:** Rhia Shah, Amol Deokar, Mahnoor Zafar, Sayak Roy, Jagdeep Singh Dagar

**Affiliations:** 1 Trauma and Orthopaedics, Princess Royal University Hospital, King's College NHS Foundation Trust, London, GBR; 2 Internal Medicine, Kings College Hospital NHS Trust, London, GBR; 3 Acute Medicine, Princess Royal University Hospital, Orpington, GBR; 4 Emergency Department, Northampton General Hospital NHS Trust, London, GBR

**Keywords:** bazett's formula, framingham formula, fridericia's formula, hodges formula, qtc interval

## Abstract

Background: Accurate measurement of the corrected QT (QTc) interval is crucial for assessing the risk of arrhythmias. Various formulas, including Bazett’s, Fridericia’s, Framingham’s, and Hodges’, are used to calculate QTc, but discrepancies between them can lead to clinical misclassification.

Objectives: This study aimed to compare the performance of different QTc formulas and identify the most reliable method for clinical practice.

Methods: A retrospective analysis was conducted on 200 type 2 diabetic patients from South-East London. QT and RR intervals were measured to calculate QTc values. Due to the non-normal distribution of QTc values (Shapiro-Wilk test, p < 0.05), non-parametric statistical methods were employed, including the Friedman test and pairwise Wilcoxon signed-rank tests, to assess differences between QTc formulas.

Results: The mean QTc values differed significantly between formulas, with Bazett’s formula producing higher values (445 ± 30 ms) compared to Fridericia’s (426 ± 29 ms), Framingham’s (424 ± 28 ms), and Hodges’ (428 ± 29 ms) formulas. The Friedman test confirmed significant variation across formulas (χ²(3) = 218.86, p < 0.001). Fridericia’s formula demonstrated consistent performance and lower variability compared to other formulas.

Conclusion: This study highlights the importance of selecting an appropriate QTc correction formula. Fridericia’s formula may be a more reliable choice for accurate QTc interval measurement in clinical practice, particularly in patients with varying heart rates.

## Introduction

The QT interval represents the time from the beginning of the QRS complex to the end of the T wave on an electrocardiogram (ECG) [1], reflecting the duration of ventricular depolarization and repolarization [2]. It is crucial to assess the QT interval in multiple ECG leads. For further assessment, the longest measurable QT interval should be considered. Leads II and V5 are usually preferred for measuring the QT interval, as they provide better visualization of the onset of the QRS complex and the end of the T wave [3].

The corrected QT interval (QTc) standardizes the QT interval to a heart rate of 60 beats per minute, and this permits a meaningful comparison of QT values through varying heart rates that aids in the identification of individuals at increased risk for arrhythmias, i.e., Torsades de Pointes [4]. Four formulas are generally employed to calculate QTc, which include the Bazett, Fridericia, Hodges, and Framingham formulae [4]. Although the most widely used is the Bazett formula, it has notable limitations, specifically, a tendency to overcorrect at higher heart rates (>90 bpm) and undercorrect at lower heart rates (<60 bpm) [5].

As per the Bazett formula, the QTc interval is considered prolonged when it exceeds 440 ms in males and 460 ms in females [6]. Both congenital and acquired prolongation of QTc due to the use of certain drugs (such as antipsychotics or antibiotics) or from an imbalance of electrolytes (that comprises hypocalcemia, hypokalemia, or hypomagnesemia) is a well-recognized risk factor that leads to the development of Torsades de Pointes (TdP), a potentially fatal ventricular arrhythmia associated with cardiovascular morbidity and sudden cardiac death [7]. Therefore, early detection and prompt management of a prolonged QTc are critical to prevent life-threatening outcomes. However, dependence merely on the Bazett formula can lead to overestimation of QTc, potentially resulting in overdiagnosis and further leading to unnecessary treatment of patients who may have a normal QTc estimated by other correction methods. Hence, to address this limitation, the current retrospective study was planned and conducted to evaluate which QTc correction formula provides the most accurate assessment and aids in avoiding overtreatment.

## Materials and methods

Study design

This was a retrospective study where data were collected from patients’ electronic medical records of two hospitals in South-East London. The data was collected between May and August 2025 and was completely anonymized so that there was no identifiable patient data.

Study population

All adult type 2 diabetes patients over 18 years of age who were admitted to surgical wards for elective minor procedures with an ECG done during their first day of admission were selected. Type 2 diabetics may have autonomic dysfunction, altered repolarization, or polypharmacy patterns that influence QT physiology. Exclusion criteria included use of insulin and intake of common medications that affect the QTc, like fluoroquinolones, macrolides, antidepressants (sertraline, duloxetine), and antipsychotics (escitalopram, haloperidol). Pregnant females, type 1 diabetes patients, and patients having electrolyte imbalances or sepsis were also excluded from the study. Patients were excluded from further analysis when not in sinus rhythm or when the QRS duration was >120 ms.

Sample size calculation

We included 200 patients in this retrospective analysis. Because all QTc correction formulae were applied to the same patients, comparisons were paired, which markedly increases statistical power. For a paired t-test at two-sided α=0.05 and 80% power, the minimum detectable mean difference (Δ) is given by:

 Δ = (Z1-α/2 + Z1-β) σd/√n

Where σd is the standard deviation of the paired differences and n the number of paired observations. With n = 200 (√n ≈ 14.1), even if the paired SD were as large as 20 ms, the minimum detectable mean difference would be ≈ 4 ms. The observed mean differences between formulae in our dataset were substantially larger (≈ 17-21 ms when comparing Bazett with other formulae). Thus, our sample of 200 patients is more than adequate to detect clinically relevant differences (≥10 ms) in QTc across correction formulae. In addition, we assessed agreement between formulae using Bland-Altman analysis. For limits of agreement, the precision of the estimate depends on the number of paired observations: the 95% confidence interval for each limit can be approximated as

LoA CI=±1.96× σd/√n​​

With 200 paired measurements, this yields a margin of error of roughly 2-3 ms for the limits of agreement, even when σd is as high as 20-30 ms. Therefore, our sample size also provides sufficient precision to characterize bias and agreement between QTc formulae.

Statistical methods

QT and RR were measured for QTc calculation. We measured the RR interval by counting the number of squares between adjacent R waves and converting this measurement to seconds. For example, multiplying the number of small squares by 0.04, or the number of large squares by 0.20, yields the RR interval in seconds. This value is then incorporated into one of several established QT-correction formulas. For irregular rhythms, particularly atrial fibrillation, we used an average of multiple consecutive five RR intervals to obtain a representative value before applying the QT-correction formula. We used values from lead II and V5.

We used machine-generated readings for those patients with normal sinus rhythm, and manual calibration for QTc estimation for those with sinus arrhythmia, tachycardia, or bradycardia. Irregular rhythms, severe bradycardia, and severe tachycardia were not included for assessment in this study.

Normality of QTc values was assessed using the Shapiro-Wilk test, which indicated that QTc values from all correction formulae deviated from a normal distribution (p < 0.05). Although skewness statistics suggested approximate symmetry, the assumption of normality required for parametric tests was not met. Therefore, we used non-parametric methods: the Friedman test to assess overall differences across formulae, followed by pairwise Wilcoxon signed-rank tests for post-hoc comparisons.

We initially calculated the values for the whole data set and then removed those having a heart rate (HR) of more than 90. The limitation on HR was introduced to minimize overcorrection when correcting the QT interval for RR in case of tachycardia, thereby minimizing outliers influencing further statistics. For the second analysis, we were left with a dataset of 160 patients whose HR was <90 bpm. We ran the same tests to see if Bazett’s formula is influenced by HR alone or not.

## Results

A total of 200 patients were included in the analysis. The median age of the cohort was 74 years, and 60% were male. Mean QTc values according to the correction formula applied were Bazett’s 445 ± 30 ms, Fridericia’s 426 ± 29 ms, Framingham’s 424 ± 28 ms, and Hodges’ 428 ± 29 ms.

A summary of all the baseline characteristics for all two hundred patients has been formulated below using the descriptive statistics calculator online in Table 1 [8].

**Table 1:** Baseline characteristics of two hundred patients HbA1c: glycated hemoglobin; eGFR: estimated glomerular filtration rate; Q1, Q3: quartiles

Groups	Age (years)	HbA1c (in %)	eGFR (mL/min/1.73 m²)	QTc – Bazett (msec)	QTc – Friedricia (msec)	QTc – Hodges (msec)
Number of observations	200	200	200	200	200	200
Minimum	40	4.5	4	357	358	369
Maximum	99	17.2	90	505	502	575
Range	59	12.7	86	148	144	206
Mean (x̄)	72.745	8.03	54.82	445.28	426.05	428.13
Standard Deviation (S)	12.54	2.23	28.20	30.07	29.22	29.28
Q1	63	6.5	30	423	405	406
Median	74	7.4	57.5	446	424	425.5
Q3	82	9.3	84	468	445.5	447
Interquartile range	19	2.8	54	45	40.5	41
Skewness	-0.28	1.33	-0.19	-0.12	0.24	0.91

**Figure 1:**
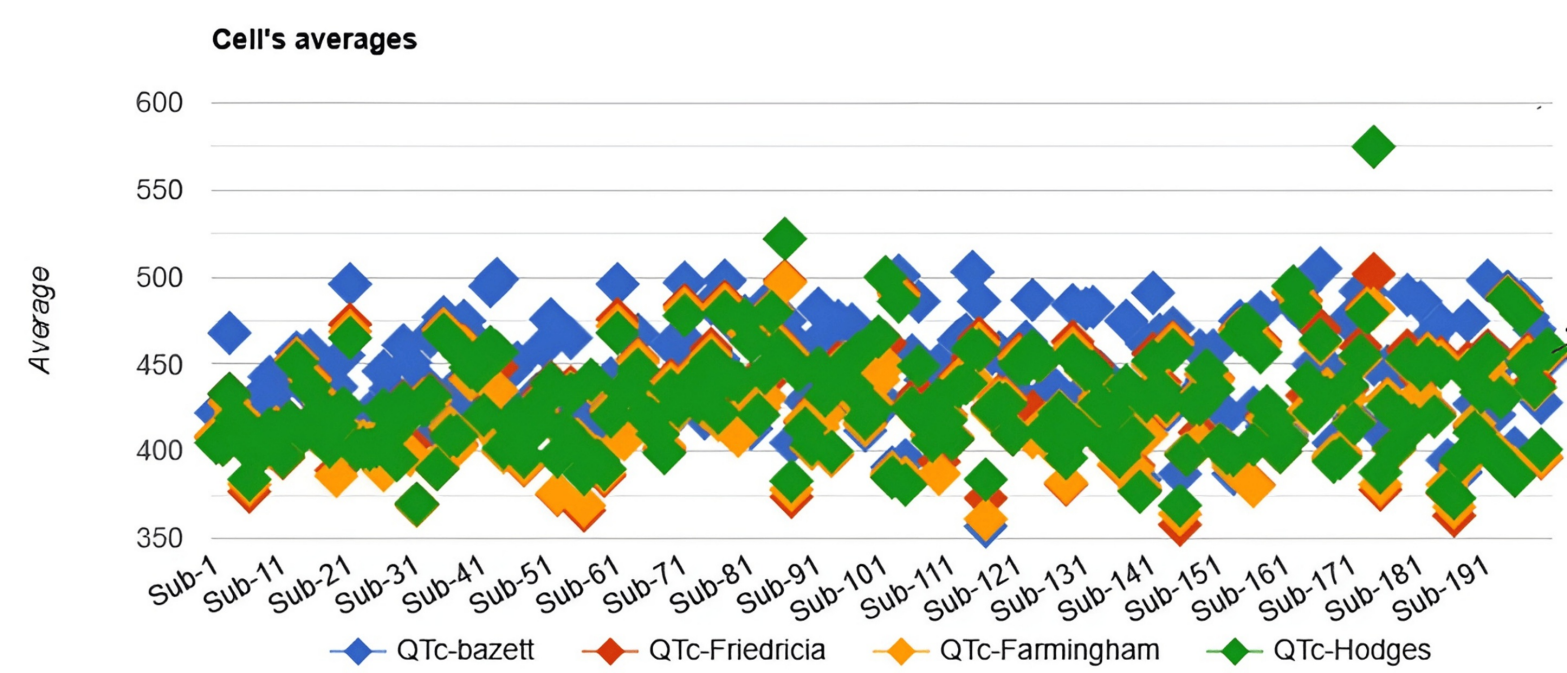
Scatter plot for all QTc values in the Friedman test for 200 patients

To assess whether QTc values differed across the four correction formulae (Bazett, Fridericia, Framingham, and Hodges), we used the Friedman test, a non-parametric test for repeated measures. The Friedman test demonstrated a statistically significant difference in QTc values across the four formulae (χ²(3) = 218.86, p < 0.001). A scatter plot of QTc values across all 200 patients (Figure 1) illustrates the systematic differences between formulae.

The plot showed Bazett consistently produced higher QTc values than Fridericia and Framingham, while Hodges' values were generally intermediate but showed occasional outliers. These visual differences correspond with the significant effect detected by the Friedman test (χ²(3) = 218.86, p < 0.001).

Because Friedman showed that a difference exists, we still needed a post-hoc pairwise comparison to find where the differences lie. That is why we ran the pairwise Wilcoxon signed-rank test between QTc-Bazett as compared with QTc values calculated by Friedricia, Farmingham, and Hodges, and it showed statistically significant values (p<0.001) between all three groups (Table 2).

**Table 2:** Wilcoxon signed-rank test between various QTc values against the QTc-Bazett value for all 200 patients BZ: Bazett; Frd: Friedricia; Frmghm: Farmingham; Hdge: Hodges

Parameter	QTc-BZ v/s Frd	QTc-BZ v/s Frmghm	QTc-BZ v/s Hdge
Z	-10.72	-11	-10.3
r	-0.8	-0.8	-0.7
p-value	<0.001	<0.001	<0.001
Number of pairs (N)	200	200	200
Non-zero difference pairs (n)	197	190	196
Skewness	0.2791	-0.4519	3.0565

**Figure 2:**
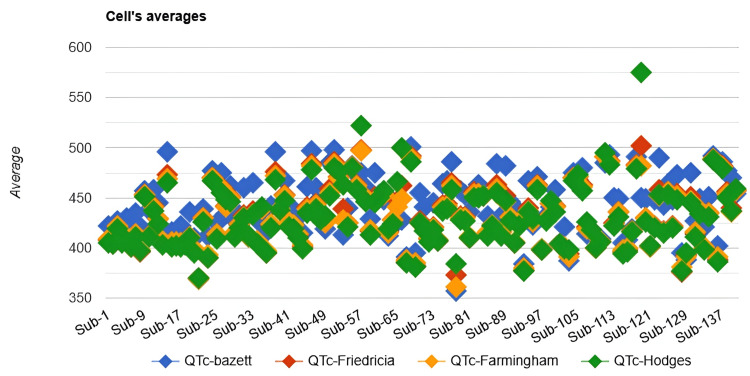
Scatter plot for all QTc values in the Friedman test of 140 patients

We found 60 patients whose HR was greater than 90 and recalculated the data using the new set (n=140) to see if the significance still existed between the QTc values of an individual, irrespective of HRs. We started again with the Friedman test, and it demonstrated a statistically significant difference in QTc values across the four formulae (χ²(3) = 152.85, p < 0.001). The effect size was moderate (Kendall’s W = 0.36), indicating a medium magnitude of difference in QTc values across correction methods. A scatter plot of QTc values across the post-excluded 140 patients (Figure 2) illustrates the systematic differences between formulae.

Bazett generally produced higher QTc values compared with Fridericia and Framingham, whereas Hodges tended to yield lower estimates but occasionally gave markedly higher outlying values.

Friedman test results, pre-exclusion and post-exclusion (excluding 60 patients’ data having HR >90 bpm), as summarized below in Table 3, show the statistical significance existed even after removing those having tachycardia.

**Table 3:** Friedman test results to assess overall differences across formulae, pre- and post-exclusion

Parameters	Data for 200 Patients	Data for 140 Patients
Test statistics (χ²)	218.86	152.85
p-value	<0.001	<0.001
Effect size (Kendall’s W)	0.36	0.36

Using the same analytical procedure, we followed up this post-exclusion cohort’s Friedman test with a Wilcoxon signed-rank test to see where the differences lie. It showed statistically significant values (p<0.001) between all three groups (Table 4).

**Table 4:** Wilcoxon signed-rank test between various QTc values against the QTc-Bazett value for 140 patients (after removing patients with HR >90 bpm) BZ: Bazett; Frd: Friedricia; Frmghm: Farmingham; Hdge: Hodges

Parameter	QTc-BZ v/s Frd	QTc-BZ v/s Frmghm	QTc-BZ v/s Hdge
Z	-7.8	-8.2	-7.5
r	-0.7	-0.7	-0.6
p-value	<0.001	<0.001	<0.001
Number of pairs (N)	140	140	140
Non-zero difference pairs (n)	137	130	136
Skewness	1.3453	0.4508	3.1778

**Figure 3:**
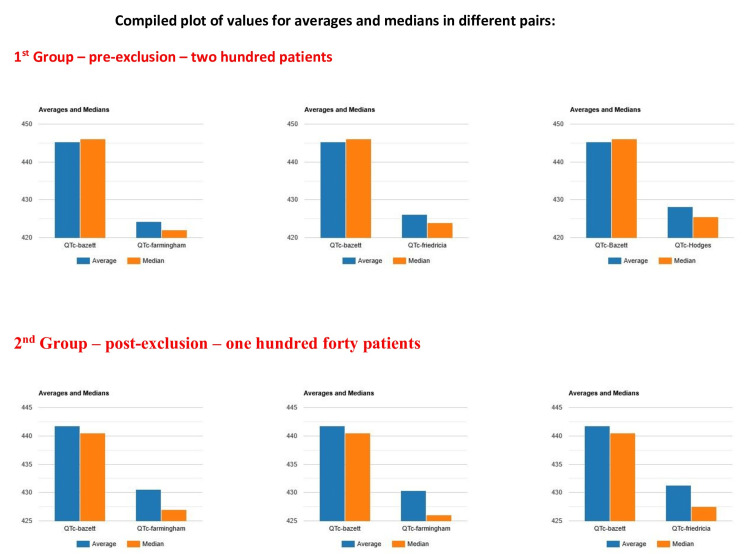
Compiled data for all averages and medians of pre- and post-exclusion patients

We have summarized the averages and median values for both the set of samples pre-exclusion and the post-exclusion group in Figure 3.

## Discussion

Prolongation of the QT interval has been associated with an increased risk for sudden cardiac death (SCD) in several population-based cohort studies [9]. The Atherosclerosis Risk in Communities (ARIC) study further tried to find if any components within the QT interval are responsible for its association with sudden cardiac death and found that the risk of SCD with the QT interval is driven by prolongation of the T wave onset to T-peak component [9]. As there are multiple formulae for calculating QTc values, selecting the best possible calculator for accurate interpretation is crucial. In lieu of this, the present retrospective analysis among 200 patients was carried out, and the study revealed a notable difference in QTc interval values based on the correction formula used. Specifically, Bazett's formula reported significantly higher QTc values compared to Fridericia’s, Framingham’s, and Hodges’ formulas. Furthermore, these differences remained consistent even after excluding patients with increased HRs, indicating that Bazett's formula's overestimation was not merely due to tachycardia. The study underscores the shortcomings of Bazett's correction, notably its propensity for overcorrection at elevated HRs and undercorrection at decreased heart rates, potentially resulting in clinical misdiagnosis and unnecessary treatment adjustments. Conversely, Fridericia's and Hodges' formulas yielded more reliable and physiologically consistent QTc estimates.

Our findings align with Vandenberk et al., who retrospectively analyzed ECGs from 6609 patients and found that the Fridericia and Framingham correction formulae had the best rate correction and significantly improved prediction of 30‐day and 1‐year mortality. It also suggested Bazett overestimated the number of patients with potentially dangerous QTc prolongation, which could lead to unnecessary safety measures by withholding the patient’s first‐choice medication and avoidable consumption of resources [10]. Similarly, a large-scale study by Andršová et al. evaluated intra-subject variability of QTc intervals across 10 different heart rate correction formulas using over 450,000 ECGs from healthy volunteers, and analysis showed that Bazett’s correction produced significantly higher short- and long-term QTc variability compared to other formulas (p < 0.00001). Fridericia and Framingham corrections demonstrated lower and comparable variability, aligning more closely with individually corrected QTc values [11]. The findings reaffirm that Bazett’s formula should be avoided in clinical practice, with Fridericia or Framingham corrections preferred for more reliable QTc monitoring.

Further, in concordance with the present study, Gueta et al. found that the mean QTc by Bazett’s formula (412 ± 20 ms) was higher than by other corrections (400 ± 16 ms). Using Bazett’s correction, 9.6% of subjects showed ΔQTc ≥ 60 ms, and 2.7% had QTc ≥ 500 ms. QTc ≥ 500 ms was significantly associated with the number of measurements (HR = 5.01, p = 0.026). The study concludes that apparent QTc prolongation may partly reflect within-individual variability, especially when using Bazett’s formula, which should be considered before modifying drug therapy [12]. Similarly, our findings are further supported by a recent study by Mkhwanazi et al. of 631 patients with diabetes, which found that Fridericia's formula outperformed Bazett’s formula in calculating QTc intervals, regardless of HIV status. This study used QTc/RR regression analysis and found that Fridericia's formula had the lowest r-squared value closest to zero, indicating minimal influence from heart rate. These results further support the use of Fridericia's formula as a reliable method for QTc calculation in diverse patient populations [13]. Another study by Aytemir et al. on 21 healthy subjects (aged 37 ± 10 years, 15 male) investigating the differences in five different formulae for heart rate correction of the QT interval in serial electrocardiograms recorded in healthy subjects subjected to graded exercise showed the Bazett, Hodges, and nomogram formulae led to significant prolongation of QTc intervals with exercise, while the Framingham formula led to significant shortening of QTc intervals with exercise [14].

Another concordant large-scale study by Luo S et al. of 10,303 normal ECGs analyzed the classical Bazett, Fridericia, Framingham, and Hodges formulas and found that normal ECGs from US hospitals showed a similar distribution of QTc obtained from the Fridericia, Framingham, and Hodges formulas, whereas Bazett’s formula showed a significantly wider distribution [15]. One of the Indian studies, conducted by Mondal H et al. on 1140 ECGs, showed the highest variance of residuals in Bazett’s formula and the lowest variance in the Framingham formula [16].

Our study echoes the same problem of having a significant statistical difference between all the formulae used to calculate QTc when we compared Bazett’s value with all the other three formulae.

Study limitations

This study has a few limitations, including its retrospective design and potential biases that come with it. The sample size, although adequate, may not be representative of the broader population. Additionally, the findings may not be generalizable to patients with specific conditions or characteristics not represented in the study population. Further research is recommended. We had the data for type 2 diabetes patients only, which cannot be generalized to the normal population. This study also had several other limitations, including not addressing β-blocker use, not accounting for diabetes duration, and not accounting for other comorbidities, such as diabetic autonomic neuropathy, that might also have influenced the QTc.

## Conclusions

This study compares QTc formulas methodologically but does not correlate them with clinical outcomes such as arrhythmias, drug changes. Our study echoes the same findings of overcorrection of the QTc value when calculated by Bazett’s formula, even after removing patients having HR >90 bpm. These findings highlight that continued reliance on Bazett risk overdiagnosis of QT prolongation, unnecessary interventions, and withholding of appropriate therapy. Fridericia and Framingham provide more reliable correction across a range of heart rates and should be preferred for clinical practice and monitoring, in agreement with previous studies.
